# Phosphorylation of RCC1 on Serine 11 Facilitates G1/S Transition in HPV E7-Expressing Cells

**DOI:** 10.3390/biom11070995

**Published:** 2021-07-06

**Authors:** Xiaoyan Hou, Lijun Qiao, Ruijuan Liu, Xuechao Han, Weifang Zhang

**Affiliations:** Department of Microbiology, School of Basic Medical Sciences, Cheeloo College of Medicine, Shandong University, Jinan 250012, China; houxiaoyan@mail.sdu.edu.cn (X.H.); lijunqiao@jnu.edu.cn (L.Q.); 201815091@mail.sdu.edu.cn (R.L.); 201915095@mail.sdu.edu.cn (X.H.)

**Keywords:** HPV, cervical cancer, RCC1, G1/S transition, phosphorylation

## Abstract

Persistent infection of high-risk human papillomavirus (HR-HPV) plays a causal role in cervical cancer. Regulator of chromosome condensation 1 (RCC1) is a critical cell cycle regulator, which undergoes a few post-translational modifications including phosphorylation. Here, we showed that serine 11 (S11) of RCC1 was phosphorylated in HPV E7-expressing cells. However, S11 phosphorylation was not up-regulated by CDK1 in E7-expressing cells; instead, the PI3K/AKT/mTOR pathway promoted S11 phosphorylation. Knockdown of AKT or inhibition of the PI3K/AKT/mTOR pathway down-regulated phosphorylation of RCC1 S11. Furthermore, S11 phosphorylation occurred throughout the cell cycle, and reached its peak during the mitosis phase. Our previous data proved that RCC1 was necessary for the G1/S cell cycle progression, and in the present study we showed that the RCC1 mutant, in which S11 was mutated to alanine (S11A) to mimic non-phosphorylation status, lost the ability to facilitate G1/S transition in E7-expressing cells. Moreover, RCC1 S11 was phosphorylated by the PI3K/AKT/mTOR pathway in HPV-positive cervical cancer SiHa and HeLa cells. We conclude that S11 of RCC1 is phosphorylated by the PI3K/AKT/mTOR pathway and phosphorylation of RCC1 S11 facilitates the abrogation of G1 checkpoint in HPV E7-expressing cells. In short, our study explores a new role of RCC1 S11 phosphorylation in cell cycle regulation.

## 1. Introduction

Cervical cancer, which is highly associated with human papillomavirus (HPV) infection, is one of the most common risk factors for women’s health [1]. HPVs are circular, non-enveloped, double-stranded DNA viruses [2] and E6, E7 of high-risk HPVs are the main viral genes responsible for cervical carcinogenesis [3]. The HPV oncogenic protein E6 induces the degradation of tumor suppressor p53 [4], while E7 binds to and degrades retinoblastoma (pRb) [5]. These two oncogenic proteins play a key role in complex cellular programs such as proliferation, differentiation, transformation and tumor maintenance [5]. The transforming activity of high-risk HPV E7 is basically mediated through protein–protein interactions [6]. However, many proteins involved in HPV E7-mediated cell cycle regulation are still undiscovered.

Regulator of chromosome condensation 1 (RCC1), a necessary guanine nucleotide exchange factor (GEF) in the nucleus for Ran GTPase, participates in cellular processes such as nuclear envelope assembly, nucleo-cytoplasmic transport and spindle formation [7,8,9,10]. RCC1 plays a crucial role in recruiting and the conversion of RanGDP into RanGTP through binding to chromatin [11]. Furthermore, chromatin transiently interacts with RCC1, which is a critical kinetic parameter for the guanine nucleotide exchange reaction [12]. The effective binding to chromatin relies on the affinity of the RanGTP complex, and promotes local Ran activation [13]. The increased expression of RCC1 is related to high cellular RanGTP levels, which accelerates cell cycle progression and controls cellular responses to DNA damage [14]. Otherwise, loss of RCC1 causes G1/S cell cycle arrest [15]. Research proved that RCC1 was up-regulated in mantle-cell lymphomas and lung adenocarcinoma tissues [16,17]. Our previous data showed that RCC1 was highly expressed in cervical cancer tissues and cells, and contributed to G1 checkpoint abrogation in HPV E7-expressing cells [18].

The phosphatidylinositol 3-kinase (PI3K)/AKT/mammalian target of rapamycin (mTOR) signaling pathway is known to be critical in proliferation, differentiation, angiogenesis, metabolism and survival [19]. At the initiation of the PI3K/AKT/mTOR signaling pathway, PI3K is divided into three types: PI3K I, PI3K II and PI3K III, among which the most concerning is PI3K I. PI3K is composed of a regulatory subunit (p85) and catalytic subunit (p110) [20,21,22]. AKT, a serine/threonine protein kinase with a wide range of substrates [23], is a major downstream target protein and signal transduction center in the PI3K/AKT/mTOR signaling pathway [24,25,26]. AKT includes AKT1, AKT2 and AKT3. AKT1 promotes cell growth, transformation and survival. AKT2 is mainly involved in the homeostasis regulation of insulin on carbohydrate metabolism. AKT3 plays an important role in regulating cell sizes and numbers [27,28,29]. There are two different mTOR complexes: mTOR complex 1 (mTORC1) and mTOR complex 2 (mTORC2) [30,31]. mTOR activation monitors changes in the external environment, cellular energy levels, oxygen content, mitotic signals, linking with cell growth and proliferation, anabolism and energy storage [32]. Numerous studies have shown the importance of the mTOR pathway in cancer pathogenesis. One research study proved that the PI3K/AKT/mTOR pathway was activated in about 70% of ovarian cancers [33]. Compared with non-tumor gastric mucosa, the mTOR pathway was activated abnormally in advanced gastric cancer [34]. A high level of mTOR has been observed in breast cancer, glioblastoma and gastric cancer [34]. AKT1 was over-expressed in gastric carcinoma [35], and AKT2 was over-expressed in ovarian and pancreatic cancer [36,37]. Thus, the PI3K/AKT/mTOR pathway is an extremely complicated intracellular network, but the precise role of the PI3K/AKT/mTOR pathway in cervical cancer is still uncertain.

In the present study, we show that phosphorylation of RCC1 S11 is up-regulated by the PI3K/AKT/mTOR pathway and contributes to G1 checkpoint abrogation in HPV E7-expressing cells. These data provide novel insights into the cellular signaling pathways that are targeted by HPV E7 and explore a new role of RCC1 S11 phosphorylation beyond the phase of mitosis.

## 2. Materials and Methods

### 2.1. Cell Culture

Spontaneously immortalized human foreskin keratinocytes (NIKS) were cultured on mitomycin C-treated J2-3T3 mouse fibroblast feeder cells in E medium composed of Ham’s F12 medium and Dulbecco’s modified Eagle medium (DMEM) (3:1) plus 5% FBS. Human telomerase reverse transcriptase-expressing human retinal pigment epithelial (RPE1) cells were maintained in a 1:1 dilution of Ham’s F12 and DMEM medium plus 10% FBS. HPV16 E7-expressing NIKS and RPE1 cells were established using a pBabe retroviral system and used within 15 passages, as described previously [38]. Cervical cancer cell lines HeLa and SiHa were maintained in DMEM. All cells were grown in medium with the addition of penicillin and streptomycin in a humidified 5% CO_2_ atmosphere at 37 °C.

### 2.2. Western Blot

Protein extracts were prepared in radio-immunoprecipitation assay (RIPA) lysis buffer, and Western blot was performed with primary antibodies against RCC1 (1:500, sc-55559, Santa Cruz, TX, USA), p-RCC1(Ser11) (1:1000, #4210, Cell Signaling, MA, USA), CDK1 (1:1000, 610038, BD Biosciences, NJ, USA), AKT (1:1000, 4961S, Cell Signaling, MA, USA), p-AKT(Ser473) (1:1000, #4060, Cell Signaling, MA, USA), p-AKT(Thr308) (1:1000, #13038, Cell Signaling, MA, USA), SP1 (1:1000, 9389s, Cell Signaling, MA, USA), p-mTOR(Ser2448) (1:1000, #5536, Cell Signaling, MA, USA), p-70S6K(Thr389) (1:1000, #5536, Cell Signaling, MA, USA), HPV16 E7 (1:300, sc1587, Santa Cruz, TX, USA), p53 (1:2000, 10442-1-AP, proteintech, Wuhan, China) and Tubulin (1:10,000, T-4026, Sigma, MO, USA). Secondary antibodies included IRDye 800CW goat anti-mouse IgG (1:10,000, 926-32210, Licor, Lincoln, NE) and IRDye 800CW goat anti-rabbit IgG (1:10,000, 926-32211, Licor, Lincoln, NE, USA).

### 2.3. Nuclear and Cytoplasmic Protein Extraction

The preparation of cytoplasmic and nuclear extracts was performed using NE-PER™ Nuclear and Cytoplasmic Extraction Reagents (Thermofisher Scientific, Waltham, MA, USA). Separation of the nuclear fraction from the cytosol was verified by immunoblotting with Tubulin (cytoplasmic) and SP1 (nuclear).

### 2.4. siRNAs and Plasmid Transfection

Cells (3 × 10^4^) were seeded on 6-cm dishes the day before transfection. Chemically modified Stealth small interfering RNAs (siRNAs) targeting RCC1, CDK1, AKT, HPV16 E6, HPV16 E7, and control siRNAs were purchased from GenePharma (GenePharma, Shanghai, China) and transfected into cells using Lipofectamine2000 (Invitrogen, Life Technologies, Carlsbad, CA, USA). Cells were transfected with siRNAs at a final concentration of 20 nM. The sequences of siRNA were as follows:

siRCC1, 5′- TGGAGATGATGGGCAAACA-3′;
siCDK1, 5′-GATCAACTCTTCAGGATTT-3′;
siAKT, 5′-GAAGGAAGUCAUCGUGGCC-3′;
E6, 5′-UACAACAAACCGUUGUGUG-3′;
E7, 5′-GCACACACGUAGACAUUCG-3′.

For rescue experiment, RCC1-expression plasmid pCMV-Flag-RCC1 (Sino Biology, Beijing, China), RCC1 S11 site mutant plasmid pCMV-Flag-RCC1 (S11A) (Sino Biology, Beijing, China) and control plasmid pCMV-Flag (Sino Biology, Beijing, China) were transfected into cells 12h after RCC1 siRNA transfection. Twenty-four hours later, cells were harvested for protein knockdown and expression analysis by Western blot.

### 2.5. Flow Cytometric (FACS) Analysis

For cell cycle analyses, cultured cells were serum-deprived for 24 h and stimulated with insulin for the indicated time. For the rescue experiment, cells were transfected with RCC1 siRNA, then transfected with RCC1 or RCC1 mutant plasmid and treated with bleomycin (Alexis Biochemicals, CA, USA) (10μg/mL in PBS) for 36 h. Cells were fixed in 70% ethanol, treated with 50 μg/mL RNase A plus 50 μg/mL propidium iodide (PI) and analyzed by flow cytometry. Cell cycle analysis was performed using Flow Jo 10 software (Becton Dickinson, NJ, USA).

### 2.6. BrdU Labeling

For bromodeoxyuridine (BrdU) labeling, BrdU (20 nM) was added to the medium 2 h before the collection of cells. Cells were then treated with BD Pharmingen™FITC BrdU Flow Kit (BD Biosciences, NJ, USA). Cells were counterstained by FITC anti-BrdU (BD Biosciences, NJ, USA) and 7-aminoactinomycin D (7-AAD) RNase A. Immuno-fluorescent cells were analyzed on a CytoFLEX 2.3 (Beckman, CA, USA).

### 2.7. Statistical Analysis

All data are presented as the means and standard deviations (SD). Statistical analyses were performed using SPSS 20.0 (SPSS, Chicago, IL, USA) and analyzed using a two-tailed Student’s *t-*test. *p* ≤ 0.05 was considered statistically significant.

## 3. Results

### 3.1. S11 of RCC1 Is Phosphorylated in HPV16 E7-Expressing Cells

**Figure 1:**
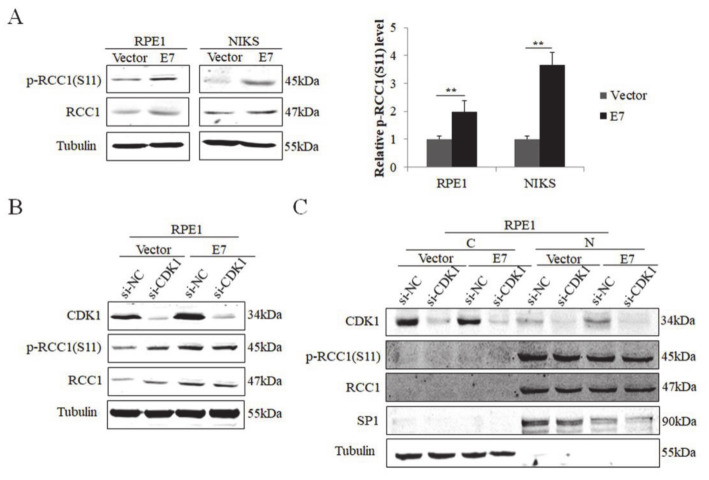
HPV E7 up-regulated phosphorylation of RCC1 S11 in CDK1-independent manner. (**A**) Western blot analysis of RCC1 and p-RCC1(S11) protein levels in HPV16 E7-expressing RPE1 and NIKS cells showed that p-RCC1(S11) was highly expressed in HPV16 E7-expressing cells. (**B**) CDK1, p-RCC1(S11), RCC1 protein levels were detected by Western blot following transfection with siRNA targeting CDK1. (**C**) Cellular localization of CDK1, p-RCC1(S11), RCC1 after transfection with siRNAs targeting CDK1 in both RPE1-Vector cells and RPE1-E7 cells. Data shown are representative of 3 biological replicates. ** p < 0.01.

The E7 oncoprotein possesses the major transforming activity of HPV. Our previous data showed that E7 up-regulated the expression of RCC1 [18]. Since RCC1 S11 phosphorylation is an important post-translational modification [39], we investigated whether HPV E7 regulates the phosphorylation of RCC1 S11. NIKS and RPE1 cells expressing control vector (Vector) or wild-type HPV16 E7 (E7) were utilized, and the expression of HPV16 E7 protein was confirmed previously [18]. Our results showed that RCC1 was highly phosphorylated on S11 in HPV E7-expressed cells compared to vector control cells (Figure 1A). Cyclin-dependent kinase 1 (CDK1) was up-regulated in E7-expressed cells [18] and was reported to phosphorylate S11 of RCC1 in mitotic human cells [39]. To verify whether E7 regulates S11 phosphorylation via CDK1, we transfected RPE1-E7 and control cells with siRNA of CDK1. CDK1 was knocked down efficiently (Figure 1B). However, RCC1 did not decrease and there was no significant change in protein levels of p-RCC1(S11) with CDK1 knockdown. We further examined the localization of RCC1 and p-RCC1(S11) proteins. The results showed that RCC1 was mainly located in the nucleus while CDK1 mostly in the cytoplasm (Figure 1C). In addition, knockdown of CDK1 had little effect on RCC1 and p-RCC1(S11) expression. These results demonstrate that HPV E7 phosphorylates RCC1 on S11 in a CDK1 non-dependent manner.

### 3.2. HPV E7 Phosphorylates RCC1 by the PI3K/AKT/mTOR Signaling Pathway

**Figure 2:**
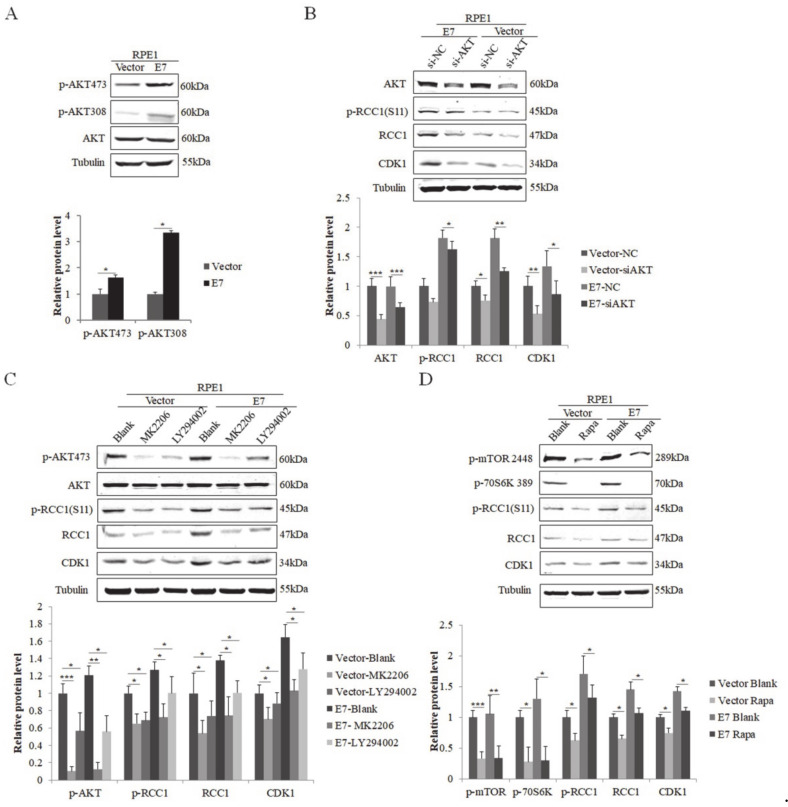
HPV E7 promoted phosphorylation of RCC1 S11 by the PI3K/AKT/mTOR pathway. (**A**) Upper panel: AKT and its phosphorylated forms were measured by Western blot. Lower panel: quantification of p-AKT 473 and p-AKT 308 proteins. (**B**) Western blot analysis of AKT, p-RCC1(S11), RCC1 and CDK1 in RPE1-E7 and control cells following AKT knockdown and quantification. (**C**) Protein levels of AKT, p-AKT 473, RCC1, p-RCC1(S11), CDK1 after treatment with MK2206 or LY294002 were detected by Western blot. (**D**) RPE1-E7 cells and control cells were treated with Rapa. As indicated, total protein lysates were analyzed for the expression of p-mTOR 2448, p-70S6K 389, p-RCC1(S11), RCC1, CDK1. Data shown are representative of 3 biological replicates. * p < 0.05; ** p < 0.01; *** p < 0.001.

The PI3K/mTOR/AKT pathway is involved in the post-translational regulation of many proteins. The phosphorylation on Ser473 and Thr308 of AKT has been used as the regulator and indicator of AKT activity [40]. Studies showed that the phosphorylation of AKT on Ser473 and Thr308 in non-small-cell lung cancer was correlated with poor prognosis [41,42]. In order to illustrate the regulation of RCC1 phosphorylation in HPV E7-expressing cells, we examined the expression of AKT and the phosphorylation of AKT on Ser473 (p-AKT 473) and Thr308 (p-AKT 308). We showed that both p-AKT 473 and p-AKT 308 increased significantly in E7-expressing cells (Figure 2A). We hypothesized that E7 promotes RCC1 phosphorylation by activating AKT. To test this idea, we used siRNA specific to AKT. Knockdown of AKT led to significant reduction in p-RCC1(S11) and CDK1 expressions in RPE1-E7 cells (Figure 2B). In addition, we used AKT pathway inhibitor MK2206 and LY294002 to treat cells. As expected, the inactivation of AKT caused a marked decrease in RCC1 and p-RCC1(S11), as well as CDK1 protein levels (Figure 2C).

As one of the downstream molecules in AKT signal, mTOR can be phosphorylated by activated AKT [43]. Meanwhile, p-70S6K, a serine/threonine protein kinase that is activated in mitosis, is vital for cell proliferation and G1/S cell cycle progression [44]. We then used rapamycin (Rapa), the specific inhibitor of mTOR, to treat cells. The decreased level of p-mTOR and p-70S6K demonstrated the effectiveness of rapamycin (Figure 2D). mTOR inhibitor treatment caused a significant decrease in RCC1, p-RCC1(S11) and CDK1 protein expressions in RPE1-E7 cells. These results suggest that the AKT/mTOR pathway promotes S11 phosphorylation of RCC1 in E7-expressing cells.

### 3.3. Phosphorylation of RCC1 S11 Occurs throughout the Cell Cycle

**Figure 3:**
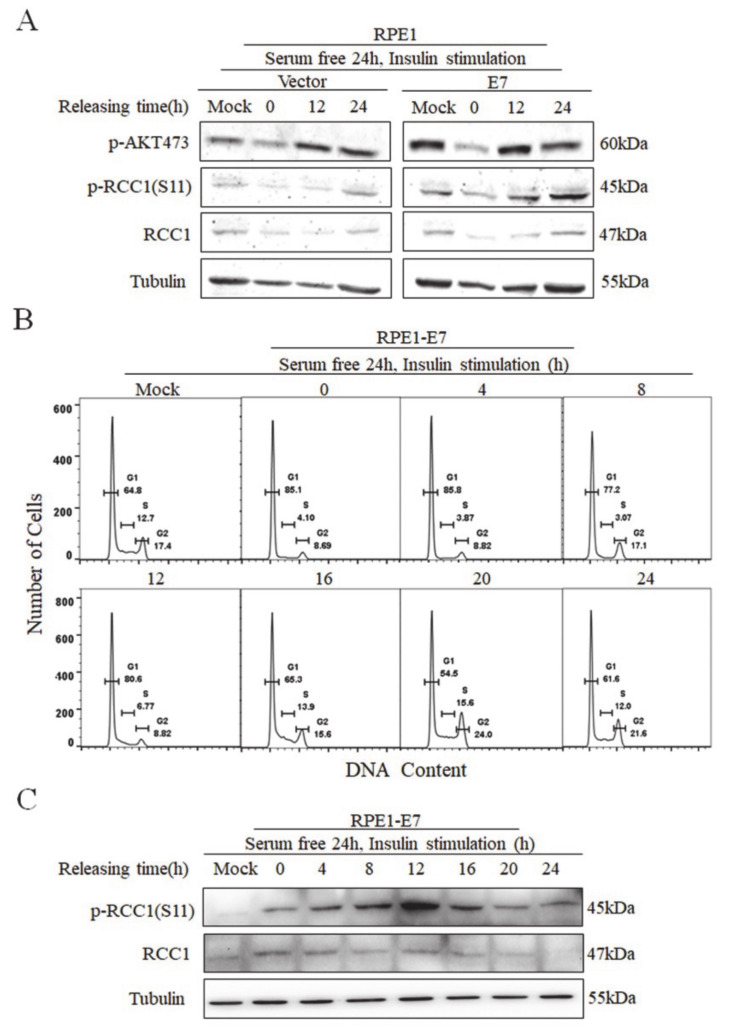
Phosphorylation of RCC1 S11 reached peak in M phase. (**A**) RPE1-E7 cells and control cells were deprived of serum for 24 h, then insulin was added, and cells were harvested at the indicated time. Expression of p-AKT 473, p-RCC1(S11), RCC1 were monitored by Western blot. (**B**,**C**) Flow cytometric and Western blot analysis of RPE1-E7 cells with serum starvation for 24 h, then insulin stimulated and harvested at different time points. Data shown are representative.

To examine the expression of S11 phosphorylation during the cell cycle, RPE1-E7 cells and control cells were serum-deprived for 24 h and stimulated with insulin. We showed that AKT was strongly phosphorylated on Ser473 at 12 h after insulin stimulation, especially in E7-expressing cells (Figure 3A). Importantly, the changes in p-RCC1(S11) level were fundamentally consistent with p-AKT, further confirming that the activation of the AKT/mTOR pathway promoted the phosphorylation of RCC1. Next, we analyzed the cell cycle of RPE1-E7 cells treated with serum starvation and insulin stimulation. Notably, serum starvation caused an obvious G1 arrest (85.1% vs. 64.8%), and insulin stimulation promoted cells, which were arrested in G1 to enter S and M phase (Figure 3B). We further detected the protein expressions of p-RCC1(S11) and RCC1 at different time points. Our results showed that RCC1 was phosphorylated as early as 0 h and increased with the progression of the cell cycle, then reached its peak at 12–16 h, which is the mitotic phase (the doubling time of RPE1 cells was approximately 20 h) (Figure 3C). After 16 h, the expression of p-RCC1(S11) gradually decreased. Since RCC1 is phosphorylated in G1 phase in E7-expressing cells, it may play a role in the G1/S transition.

### 3.4. Non-Phosphorylation of RCC1 on S11 Inhibits the G1/S Transition

**Figure 4:**
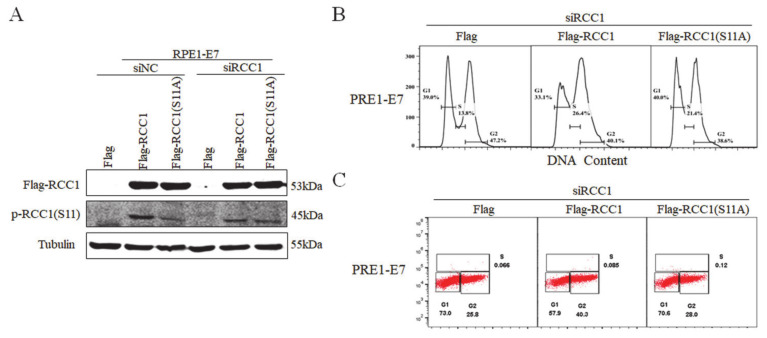
Phosphorylation of RCC1 S11 promoted G1/S cell cycle progression. (**A**) RCC1 were first knocked down with siRNA for 12 h in RPE1-E7 cells, then transfected with Flag, Flag-RCC1 or Flag-RCC1(S11A) plasmid for 12 h. The expression of p-RCC1(S11) and Flag-RCC1 proteins were measured by Western blot. (**B**) Flow cytometric analysis of cells mentioned in (**A**) with bleomycin treatment for 36 h. (**C**) BrdU incorporation analysis of cells mentioned in (**A**) with bleomycin treatment for 36 h. Data shown are representative of 3 biological replicates.

Our previous study proved that RCC1 promoted the G1/S progression [18] and we showed that the S11 of RCC1 was highly phosphorylated in HPV E7-expressing cells. In order to investigate whether S11 phosphorylation affected the G1/S transition, S11 was substituted by alanine (S11A), a mutant which mimics the non-phosphorylated status of the S11 residue. We first transfected RPE1-E7 cells and control cells with siRNA of RCC1, then overexpressed Flag, Flag-RCC1 or Flag-RCC1 S11A plasmid. Overexpression of RCC1 restored the protein level of p-RCC1(S11), but overexpression of RCC1 S11A did not restore p-RCC1(S11) in RPE1-E7 cells (Figure 4A). Consistently, flow cytometric analysis showed that RCC1 overexpression abrogated G1 arrest (33.1% vs. 39.0%) and facilitated cells to enter S phase (26.4% vs. 13.8%) when treated with DNA-damaging agent bleomycin, while the S11A mutant reduced the ability of RCC1 to rescue G1 arrest (40.0% vs. 39.0%) (Figure 4B). We measured BrdU incorporation and found that RCC1 knockdown caused E7 cells to arrest in G1 phase when treated with bleomycin, while overexpression of RCC1 rescued G1 arrest (57.9% vs. 73.0%) (Figure 4C). However, the RCC1 S11A mutant lost G1 checkpoint abrogation function (70.6% vs. 73.0%). These results indicate that phosphorylation of RCC1 S11 is necessary for the G1/S cell cycle progression.

### 3.5. S11 of RCC1 Is Phosphorylated by the PI3K/AKT/mTOR Pathway in HPV-Positive Cervical Cancer Cells

**Figure 5:**
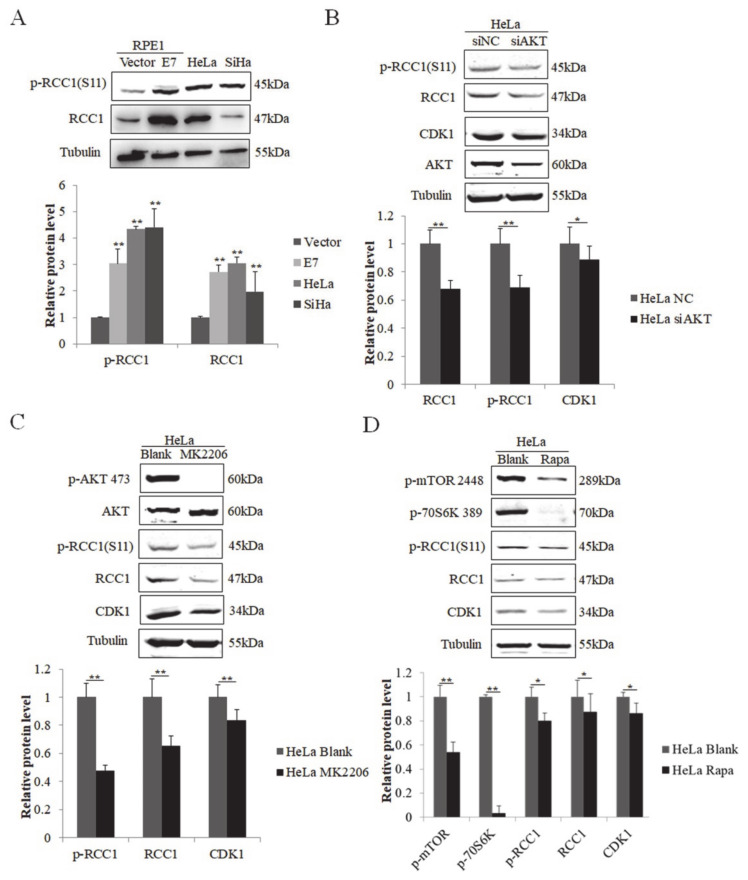
Phosphorylation of RCC1 S11 was increased by the PI3K/AKT/mTOR pathway in HeLa cells. (**A**) RCC1 and p-RCC1(S11) protein levels in RPE1-Vector, RPE1-E7, HeLa and SiHa cells were detected by Western blot. (**B**) Expression of RCC1, p-RCC1(S11), CDK1, AKT in HeLa cells after AKT knockdown. (**C**) Western blot analysis of p-AKT 473, AKT, RCC1, p-RCC1(S11), CDK1 in HeLa cells treated with MK2206. (**D**) HeLa cells were treated with Rapa, and p-mTOR 2448, p-70S6K 389, RCC1, p-RCC1(S11), CDK1 protein levels were measured by Western blot. Data shown are representative of 3 biological replicates. * *p* < 0.05; ** *p* < 0.01.

**Figure 6:**
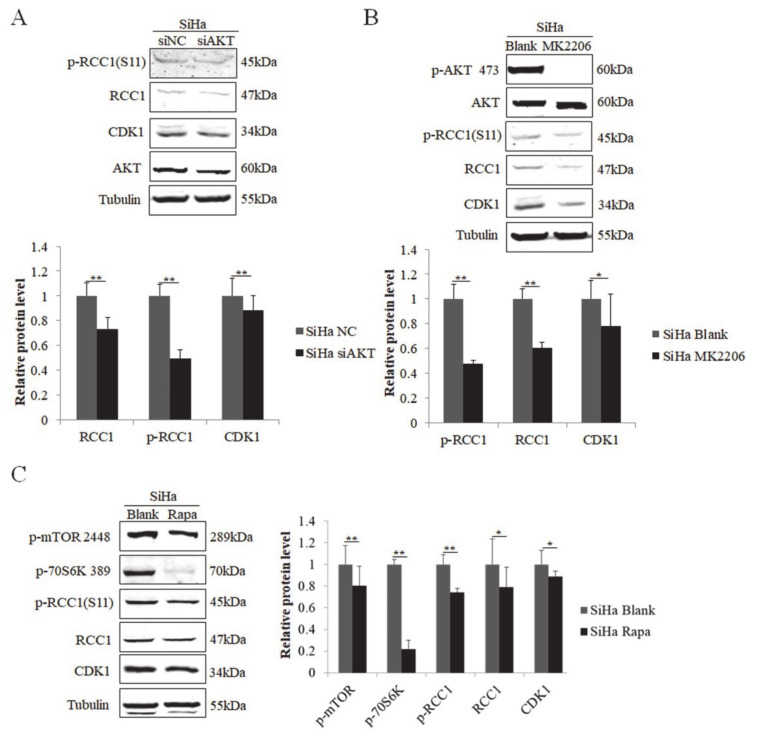
Phosphorylation of RCC1 S11 was increased by the PI3K/AKT/mTOR pathway in SiHa cells. (**A**) Expressions of RCC1, p-RCC1(S11), CDK1, AKT in SiHa cells with AKT knockdown was measured by Western blot. (**B**) Western blot analysis of p-AKT 473, AKT, p-RCC1(S11), RCC1, CDK1, in SiHa cells treated with MK2206. (**C**) p-mTOR 2448, p-70S6K 389, p-RCC1(S11), RCC1, CDK1 protein levels in SiHa cells treated with Rapa were measured by Western blot. Data shown are representative of 3 biological replicates. * *p* < 0.05; ** *p* < 0.01.

We next examined the regulation of RCC1 phosphorylation in cervical cancer cells. We showed that the protein levels of RCC1 and p-RCC1(S11) were up-regulated in HPV-positive cervical cancer HeLa and SiHa cells (Figure 5A). We then knocked down AKT in HeLa cells, and the expression of RCC1, p-RCC1(S11), and CDK1 all decreased (Figure 5B). Next, HeLa cells were treated with AKT inhibitor MK2206, and we showed that MK2206 inhibited RCC1, p-RCC1(S11) and CDK1 protein expressions (Figure 5C). Additionally, mTOR inhibitor treatment caused a significant decrease in CDK1, RCC1 and p-RCC1(S11) protein expressions in HeLa cells. We further detected the expression of RCC1 and p-RCC1(S11) in cervical cancer SiHa cells with AKT knockdown, MK2206, or rapamycin treatment and similar results were observed (Figure 6A–C). These results suggest that the AKT/mTOR pathway up-regulates RCC1 expression and promotes S11 phosphorylation in cervical cancer cells.

We further used the siRNA technology to knock down the expression of HPV16 E7 or E6 (as a control) in cervical cancer SiHa cells. As shown in Supplementary Figure S1, the decrease in HPV16 E7 protein proved that E7 was efficiently knocked down, and the increase in p53 protein proved that E6 was efficiently knocked down. We showed that the expression of RCC1 protein was reduced with both E6 and E7 knockdown. Additionally, both E6 siRNA and E7 siRNA inhibited the phosphorylation of RCC1 S11. We speculate that E7 activates the AKT/mTOR pathway, and the latter promotes RCC1 S11 phosphorylation in cervical cancer cells. Therefore, knockdown of E7 inactivates the AKT/mTOR pathway, which results in the decreased phosphorylation of RCC1 S11.

## 4. Discussion

RCC1 is involved in mammalian chromosome condensation and, as a guanine nucleotide exchange factor for Ran, the localization of RCC1 to chromosomes is necessary for the fidelity of mitosis in human cells [45,46]. Previous studies showed that phosphorylated RCC1 preferentially interacted with mitotic chromatin, which played an important role in cellular function [45], spindle assembly and chromosome segregation [46]. In human cells, RCC1 can be highly phosphorylated on serine 11 by CDK1 in mitosis [39,46]. We showed that RCC1 was strongly phosphorylated on S11 in HPV E7-expressing cells and cervical cancer cells. Furthermore, we demonstrated that S11 phosphorylation occurred as early as G1 phase and was expressed throughout the cell cycle, suggesting that S11 phosphorylation may play a role beyond mitosis.

The development of HPV-positive cervical cells relied on the sustained expression of the viral oncogenes [47]. An erroneous activation of the PI3K/AKT/mTOR pathway was observed in various types of cancers and induced malignant growth and therapy resistance [48]. Evidence revealed that the interaction between the PI3K/AKT/mTOR signaling and the HPV oncogene expression was highly complex [49]. Both the HPV E6 and E7 oncogenes were reported to activate the PI3K/AKT/mTOR signaling pathway [50,51,52]. PI3K signaling was shown as significant for HPV-induced cellular transformation [53] and HPV oncogenes were able to activate this pathway [50,51,52]. Moreover, the prognosis of cervical cancer patients was associated with the status of the PI3K/AKT/mTOR pathway. High levels of phosphorylated AKT [54] and activated mTOR [55] were associated with a poor prognosis and shorter survival. AKT phosphorylation was observed in many tumor cells [56]. However, the exact role of the PI3K/AKT/mTOR activation in RCC1-induced cervical cancer development is still unclear. Our research explored the relationship between RCC1 phosphorylation and the PI3K/AKT/mTOR pathway, and we showed that HPV E7 promoted S11 phosphorylation by activating the PI3K/AKT/mTOR signaling. Hence, our study may provide a potential target for the treatment of cervical cancer.

Cell cycle progression is monitored by several checkpoints, including G1/S, G2/M and postmitotic G1 checkpoints [57,58]. CDK1, also named cell division control protein 2 (cdc2), mediates and induces the transition from the G2 phase into mitosis [59]. Additionally, a previous study showed that CDK1 substituted for CDK2 during the G1/S transition [60]. Consistently, our laboratory showed that CDK1 acted a vital role in G1/S checkpoint, determining whether cells entered S phase and initiated DNA replication [61]. As cancer cells often display enhanced CDK1 activity, CDK1 is frequently proposed as a tumor specific marker. Moreover, one study demonstrated that CDK1 binds to many cyclins and drives the whole cell cycle progression [62]. Since CDK1 was reported to phosphorylate RCC1 in mitosis [39], we examined whether RCC1 phosphorylation was regulated by CDK1 in HPV E7-expressing cells. However, our data showed that CDK1 did not phosphorylate RCC1 on S11. This suggests that CDK1-regulated RCC1 phosphorylation may be cell type-dependent and the underlying mechanism needs to be further explored.

In the present study, we first verified that S11 phosphorylation of RCC1 increased in HPV E7-expressing cells. Next, we proved the phosphorylation of RCC1 S11 was up-regulated by the PI3K/AKT/mTOR signaling pathway instead of CDK1. S11 phosphorylation occurred not only in mitosis but also in other phases of the cell cycle. Disruption of phosphorylation of RCC1 by S11A mutation lost the ability of RCC1 to facilitate G1/S transition in E7-expressing cells. We further confirmed the expression of RCC1 S11 phosphorylation and its regulation in HPV-positive cervical cancer SiHa and HeLa cells. Taken together, this study reveals a novel function of phosphorylation of RCC1 S11 in high-risk HPV E7-mediated G1/S cell cycle progression and helps to better understand the molecular basis of HPV-associated carcinogenesis.

## Data Availability

All data presented in this work are included in the article.

## Supplementary Materials

The following are available online at https://www.mdpi.com/article/10.3390/biom11070995/s1, Figure S1: Phosphorylation of RCC1 S11 was decreased with HPV E7 knockdown.
