# Supplementary material for: Phosphorylation of RCC1 on Serine 11 Facilitates G1/S Transition in HPV E7-Expressing Cells
Source: Biomolecules. 2021 Jul 6;11(7):995. doi: 10.3390/biom11070995 (PMC8301946; doi:10.3390/biom11070995)
Supplement: Supplementary file 1 [file biomolecules-11-00995-s001.zip › biomolecules-1240877-supplementary.pdf]

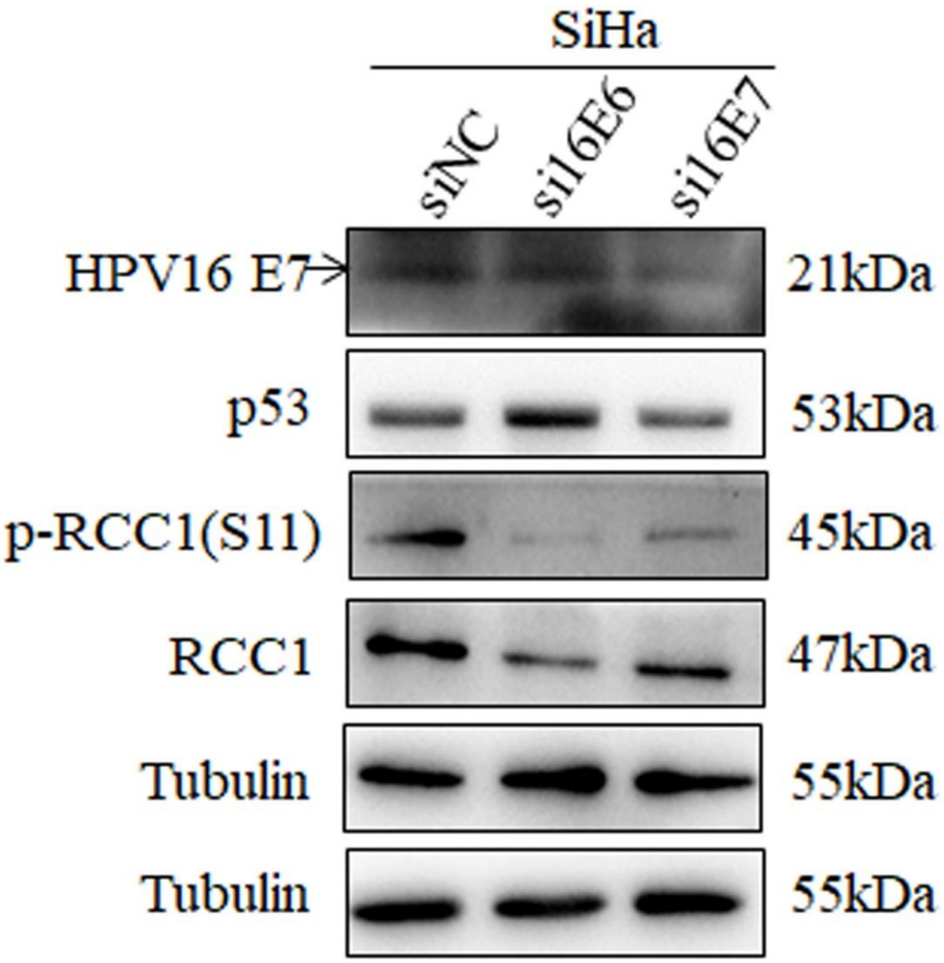

Figure S1: The protein levels of HPV16 E7, p53, p-RCC1(S11), RCC1 were detected by Western blot following transfection with siRNAs targeting HPV16 E6 or E7.
